# Enhanced Rate Capability in B-Site High-Entropy Perovskite Oxide Ceramics: The Case of La(Co_0.2_Cr_0.2_Ni_0.2_Ga_0.2_Ge_0.2_)O_3_

**DOI:** 10.3390/ma18173966

**Published:** 2025-08-25

**Authors:** Boon-How Mok, Tengfa Yao, Longchao Fu, Cheng-Tsung Lu, Haoxian Ouyang, Zongying Pan, Changan Tian

**Affiliations:** School of Chemistry and Civil Engineering, Shaoguan University, Shaoguan 512005, China

**Keywords:** high-entropy oxides, perovskite, specific capacity, electrochemical property

## Abstract

This study employed the solid-state method to prepare perovskite-type high-entropy oxide materials La(Co_0.2_Cr_0.2_Fe_0.2_Mn_0.2_Ni_0.2_)O_3_ and La(Co_0.2_Cr_0.2_Ni_0.2_Ga_0.2_Ge_0.2_)O_3_ with equimolar ratios at the B-site and explored the effects of sintering temperature on the phase structure and electrochemical properties of high-entropy oxide ceramics. The results show that after sintering at 1300°C, both samples exhibit orthorhombic perovskite structures. Both have a relative density of >97%, while La(Co_0.2_Cr_0.2_Ni_0.2_Ga_0.2_Ge_0.2_)O_3_ has a significantly larger grain size. Using these materials as electrodes, the cyclic voltammetry (CV) and galvanostatic charge–discharge (GCD) results indicate that the working electrode made of La(Co_0.2_Cr_0.2_Ni_0.2_Ga_0.2_Ge_0.2_)O_3_ shows higher oxidation reaction activity in CV measurements and achieved a specific capacitance of 74.3 F/g at a current density of 1 A/g in GCD measurements, which still maintained 73% of its initial specific capacitance (54.3 F/g) when the current density was increased to 10 A/g. Its capacitance retention rate is 10 percentage points higher than that of La(Co_0.2_Cr_0.2_Fe_0.2_Mn_0.2_Ni_0.2_)O_3_ at high current densities, demonstrating superior rate performance.

## 1. Introduction

Driven by the urgent demand for energy storage and conversion technologies, the research and development of new functional materials has always been the core driving force for breaking through technical bottlenecks. The emergence of high entropy alloys (HEAs) has initiated a brand new paradigm for the design of multi-principal element materials [1,2,3]. Through the complex solid solution structure stabilized by high mixing entropy, they exhibit excellent properties that are difficult for traditional alloys to match. Inspired by this, high entropy ceramic materials, especially high entropy oxides (HEO) [4], have become one of the research frontiers in the field of materials science in recent years, showing huge application potential in key fields such as solid-state electrolytes [5], catalytic electrodes [6], energy storage devices [7], etc.

In 2015, Rost et al. [4] first extended the concept of high-entropy alloys to the oxide system, successfully synthesizing five rock-salt-structured high-entropy oxides composed of equimolar transition metals, which laid the research foundation for high-entropy oxides. Since then, teams such as Djenadic et al. [8] and Berardan et al. [9] have successively developed rare-earth-based, lithium-based, and spinel-type high-entropy oxides [10,11], continuously enriching the compositional design and structural diversity of this system. With the deepening of research, high-entropy oxides have gradually revealed their unique advantages in the fields of electricity, catalysis, and magnetism [12,13,14,15,16,17] due to properties such as electron structure modulation induced by lattice distortion and enrichment of surface active sites, becoming an important direction to break through the performance limits of traditional single-component materials.

Perovskite-type (ABO_3_) oxides have become one of the ideal carriers for high-entropy ceramic design due to their flexible component tunability and rich physicochemical properties. In this structure, 12-coordinated A-site cations (such as rare earth/alkaline earth metals) and 6-coordinated B-site cations (such as transition metals/metalloids) are connected through oxygen octahedrons to form a highly designable crystal framework. When multi-principal cations (such as more than four elements in the A-site or B-site) are introduced, the difference in atomic size and charge imbalance will cause the tolerance factor (*t*) to deviate from the ideal value (0.8–1.0), leading to lattice distortion and reduced symmetry, thereby activating effects such as oxygen vacancy formation and electron spin state regulation [18]. These effects provide a structural basis for the high ionic conductivity and excellent redox reversibility required for electrochemical energy storage.

In 2017, Sarkar et al. [19] successfully synthesized the first perovskite-type HEO using nebulized spray pyrolysis (NSP) technology. Their research demonstrated that the introduction of multiple cations at the A-site can effectively modulate the electronic structure and lattice parameters of the B-site. This innovative approach provided a novel strategy for precisely tailoring the electromagnetic properties of materials. In 2018, Jiang et al. [20] synthesized a high-entropy perovskite oxides containing 13 different cations, thereby transcending the compositional constraints of conventional perovskites. Their comprehensive study elucidated the quantitative relationships between atomic size differences, mixing entropy, and lattice stability.

Despite significant progress in studying the composition–structure–property relationships of high-entropy perovskite oxides, the complexity of multi-principal-element synergistic effects still makes it difficult to resolve the action mechanisms of individual components. To address this challenge, this study selects a typical B-site high-entropy perovskite oxides La(Co_0.2_Cr_0.2_Fe_0.2_Mn_0.2_Ni_0.2_)O_3_ (HEO1) [21] as the benchmark. By replacing partial transition metals (Fe/Mn) with metal/metalloid elements (Ga/Ge), an isostructural La(Co_0.2_Cr_0.2_Ni_0.2_Ga_0.2_Ge_0.2_)O_3_ (HEO2) is designed and prepared. Through comparing the phase evolution, microstructure, and electrochemical behavior of these two, the influence rules of component electronegativity and ionic radius differences on the lattice stability, charge transport characteristics, and redox activity of perovskites are revealed, providing experimental basis and theoretical support for the targeted optimization of high-entropy ceramics in battery electrode materials.

Specifically, the B-site of HEO1 consists of transition metal ions including Co^2+^, Cr^3+^, Fe^3+^, Mn^3+^, and Ni^2+^; the diversity of their d-electron configurations endows the material with rich redox activity. HEO2, by introducing metalloid ions like Ga^3+^ and Ge^4+^, maintains the perovskite structure while regulating the average electronegativity and coordination number of the B-site, which is expected to optimize the reaction kinetics at the electrode/electrolyte interface. In this study, both materials were prepared via solid-state reactions. Combined with XRD, SEM/EDS, and electrochemical tests, the effects of component substitution on crystal structure, microstructural densification behavior, and rate performance are systematically analyzed, providing an example for the in-depth exploration of the “composition–structure–property” correlation mechanism in high-entropy materials.

## 2. Materials and Methods

In this study, perovskite-type high-entropy oxide ceramics were prepared by using a traditional solid-state reaction. First, analytical grade lanthanum oxide (La_2_O_3_), cobalt tetroxide (Co_3_O_4_), chromium oxide (Cr_2_O_3_), iron oxide (Fe_2_O_3_), manganese dioxide (MnO_2_), nickel oxide (NiO), gallium oxide (Ga_2_O_3_), germanium oxide (GeO_2_), titanium dioxide (TiO_2_), etc., were weighed accurately according to the stoichiometric ratio and placed in a ball-milling jar. The mixture was ball-milled at 200 rev/min for 6 h using a planetary ball mill machine. The obtained precursor was pre-sintered at 400 °C for 24 h to yield La(Co_0.2_Cr_0.2_Fe_0.2_Mn_0.2_Ni_0.2_)O_3_ (HEO1) and La(Co_0.2_Cr_0.2_Ni_0.2_Ga_0.2_Ge_0.2_)O_3_ (HEO2) powder materials. Subsequently, the pre-sintered powder was ground, and a 5% (mass fraction) polyvinyl alcohol (PVA) binder was added for uniform granulation. Approximately 0.4 g of the powder was dry pressed into a form of thin pellets with a diameter of 10 mm and a thickness of 1.5 mm under a pressure of 10 MPa. Finally, the pellets were heated to 400 °C at a rate of 5 °C/min and kept at this temperature for 24 h to slowly remove the binder. It was then heated to 1000–1400°C at a rate of 10 °C/min, held for (3–6) h, and cooled to room temperature at 10 °C/min to obtain the high-entropy oxide ceramics HEO1 and HEO2.

For crystal structure and materials composition analysis, a Smart Lab X-ray diffractometer (Rigaku, Tokyo, Japan) was used with Cu Kα radiation (wavelength λ=0.154 nm). The step size and scanning speed are 0.02° and 5°/min, respectively, over the 2θ angular range between 10° and 80°. The true density and relative density of high-entropy oxide ceramics were measured by Archimedes’ water displacement method. For morphological characterization, a SU8010 field-emission scanning electron microscope (FE-SEM) (Hitachi, Tokyo, Japan) was employed, and elemental distribution was analyzed by energy-dispersive X-ray spectroscopy (EDS).

For electrode fabrication, both ceramic powders calcined at 1300 °C were used as electrode materials, mixed with acetylene black and PVDF (0.1 g/L, dissolved in N-methylpyrrolidone) at a mass ratio of 15:2:3 to form a slurry. The slurry was coated on a nickel foam (1 cm × 1 cm) and dried at 60 °C for 3 h in a blast oven. The dried electrode was pressed at 10 MPa for ≥5 s to prepare the working electrode. Electrochemical measurements were performed using a CHI760E electrochemical workstation (Chenhua, Shanghai, China), on which cyclic voltammetry (CV) tests were conducted in a voltage window of (0–0.5) V at scan rates of (10–100) mV/s, and the Galvanostatic charge–discharge (GCD) tests were conducted at current densities of (1–10) A/g. A three-electrode system was adopted, with a saturated calomel electrode as the reference electrode, a platinum sheet (2 cm × 2 cm) as the counter electrode, and 1 mol/L KOH solution as the electrolyte.

## 3. Results

### 3.1. Phase and Morphology Characterization of La(Co_0.2_Cr_0.2_Fe_0.2_Mn_0.2_Ni_0.2_)O_3_ and La(Co_0.2_Cr_0.2_Ni_0.2_Ga_0.2_Ge_0.2_)O_3_

**Figure 1:**
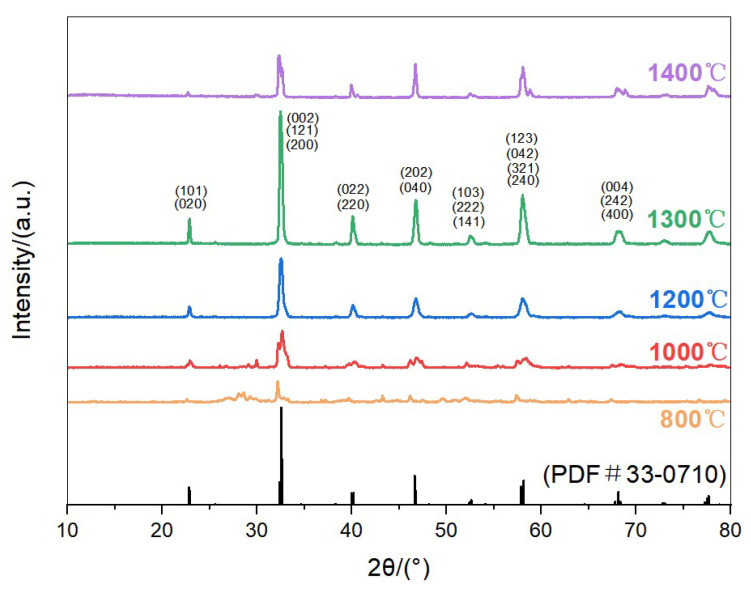
XRD patterns of La(Co_0.2_Cr_0.2_Fe_0.2_Mn_0.2_Ni_0.2_)O_3_ (HEO1) high-entropy oxide ceramics calcined at different temperatures.

**Figure 2:**
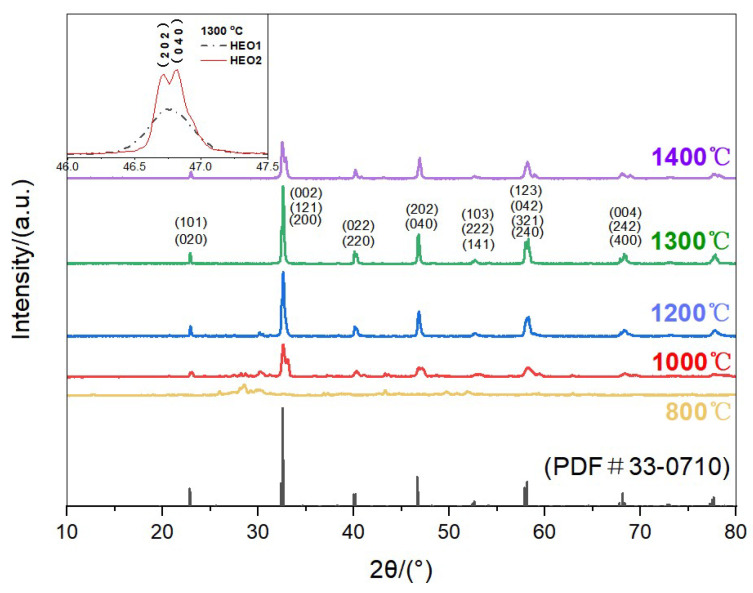
XRD patterns of La(Co_0.2_Cr_0.2_Ni_0.2_Ga_0.2_Ge_0.2_)O_3_ (HEO2) high-entropy oxide ceramics calcined at different temperatures.

Figure 1 and Figure 2 show the XRD patterns of La(Co_0.2_Cr_0.2_Fe_0.2_Mn_0.2_Ni_0.2_)O_3_ (HEO1) and La(Co_0.2_Cr_0.2_Ni_0.2_Ga_0.2_Ge_0.2_)O_3_ (HEO2) high-entropy ceramic powders calcined at different temperatures for 3 hours. The crystal structure evolution of HEO1 and HEO2 high-entropy oxide ceramic materials was characterized by X-ray diffraction (XRD). The results indicate that the calcination temperature is a key parameter for regulating their phase composition and crystallization quality. When the calcination temperature was 1000 °C, the initial formation of the perovskite structure was observed in both materials, but impurity-phase peaks (such as oxides or secondary phases) still existed in the diffraction patterns, suggesting that the formation of the perovskite phase was incomplete, and the crystallization process was in a thermodynamically non-equilibrium state. As the temperature increased to 1300 °C, the XRD patterns showed that the impurity phase peaks completely disappeared, and the characteristic diffraction peaks significantly increased in intensity with narrowed full width at half maximum (FWHM), indicating that both samples formed a single perovskite structure with excellent crystallinity and high-lattice integrity. Further increasing the temperature to 1400 °C led to the reappearance of impurity phase peaks, implying that excessively high temperature caused the decomposition of the perovskite structure or the precipitation of secondary phases, which is closely related to the thermal stability window of perovskite materials [19,20]. According to the standard card (PDF#33-0710), the diffraction peak positions of HEO1 and HEO2 are highly consistent with the orthorhombic LaCrO_3_ structure, indicating that both HEO1 and HEO2 exhibit an orthorhombic crystal structure, which is consistent with the structural characteristics of high-entropy perovskite oxides in similar studies [21]. The inset of Figure 2 shows the comparison of the (2 0 2) and (0 4 0) peaks of HEO1 and HEO2 sintered at 1300 °C: the two peaks of HEO1 overlap, while those of HEO2 are clearly separated, indicating that the substitution of Fe/Mn by Ga/Ge leads to differences in lattice constants between the two oxides and changes in the elemental occupation of atomic positions.

**Figure 3:**
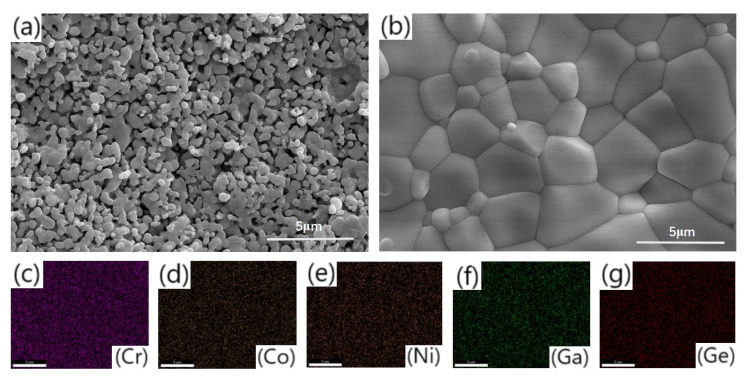
SEM image of sample HEO1 calcined at 1300 °C (**a**), SEM image (**b**) and corresponding EDS element mapping (**c**–**g**) of sample HEO2 calcined at 1300 °C.

Figure 3a,b shows the scanning electron microscope (SEM) microstructures of HEO1 and HEO2 perovskite high-entropy oxide ceramics calcined at 1300 °C. All samples were surface gold-plated to enhance conductivity, ensuring the resolution and accuracy of SEM imaging. Observations indicate that the HEO1 sample exhibits a uniform fine-grained structure with a concentrated grain size distribution, while the HEO2 sample features significantly larger and interconnected grains with clear grain boundaries and a clean, defect-free surface. This difference is attributed to the substitution effect of transition metals with metalloid and metal elements in HEO2 [1]. Energy-dispersive X-ray spectroscopy (EDS) elemental distribution analysis of the HEO2 sample (Figure 3c–g) shows that all elements are uniformly and diffusely distributed without obvious elemental segregation or agglomeration. Such atomic-scale uniformity directly reflects the “cocktail effect” of high-entropy materials—the synergistic effect of multi-principal elements suppresses elemental segregation during high-temperature sintering through an entropy stabilization mechanism, ensuring atomic-level mixing of each component in the crystal structure [8]. Since the mapping results in Figure 3 are not clear enough, we will present the original mapping files in the Supplementary Materials for reference, where Figure S1 corresponds to Figure 3c, Figure S2 to Figure 3d, Figure S3 to Figure 3e, Figure S4 to Figure 3f, and Figure S5 to Figure 3g. This provision of original files aims to supply more distinct visual data to understand the research findings.

**Figure 4:**
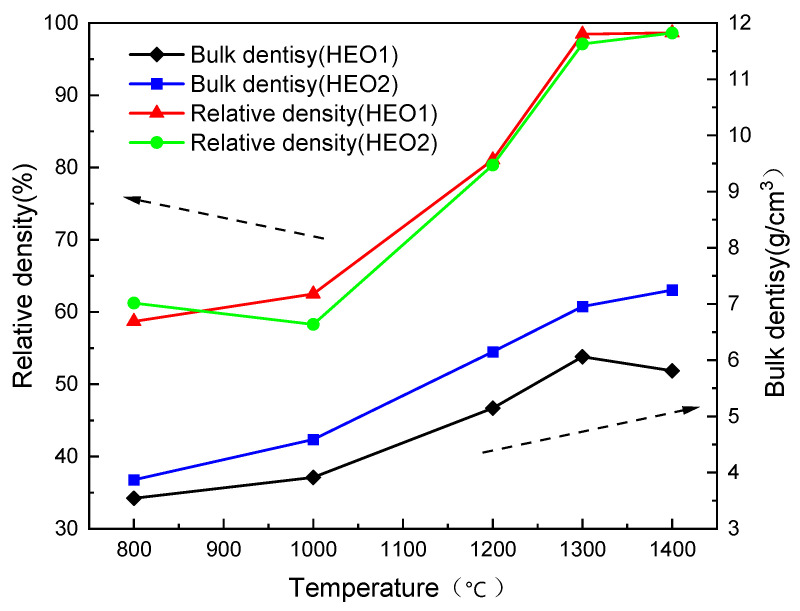
Variation of bulk density and relative density of high entropy oxide ceramics HEO1 and HEO2 sintered at different temperatures.

Figure 4 presents the evolution of density and relative density of HEO1 and HEO2 high-entropy oxide ceramic with the sintering temperature. The data show that the bulk density and densification processes of both materials follow the typical sintering kinetic characteristics: as the temperature gradually increases from the low-temperature region, the mass transfer between particles (such as grain boundary diffusion and volume diffusion) is gradually enhanced, leading to the reduction of porosity inside the bulk and the continuous increase in relative density. When the temperature exceeds 1300 °C, the relative density of all samples is higher than 97% and tends to a stable plateau, meeting the strict requirements for the compactness of electrode materials in electrochemical energy storage devices. A high relative density (>97%) means that the material has lower grain boundary resistance and shorter ion transport paths, which are crucial for improving the conductivity and cycle stability of battery materials.

### 3.2. Electrical Property Characterization

**Figure 5:**
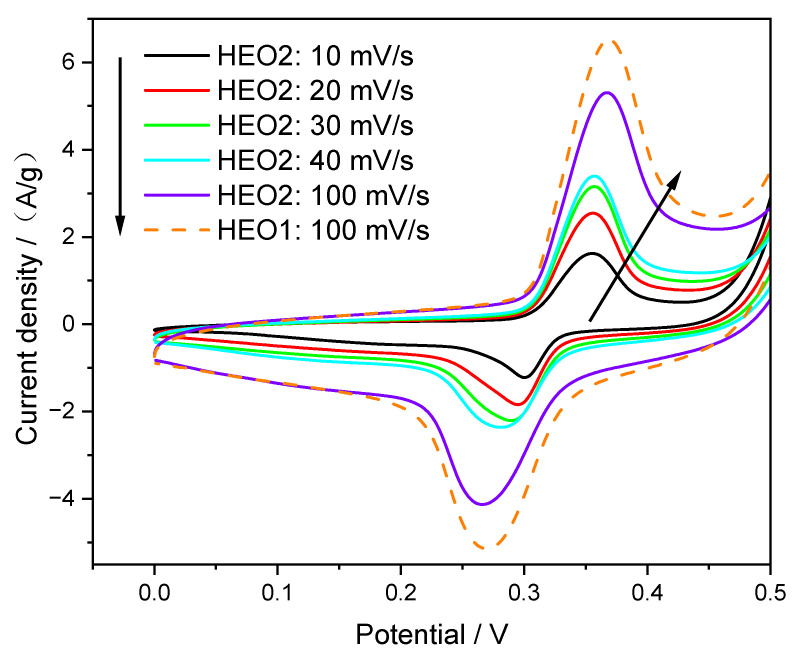
CV curves of HEO1 and HEO2 electrodes at different scan rates.

To investigate the electrical properties of the two high-entropy ceramic materials, HEO1 and HEO2, cyclic voltammetry (CV) and galvanostatic charge–discharge (GCD) tests were conducted on the prepared working electrodes. As shown in Figure 5, the CV curve of the HEO1 sample (indicated by the dashed line) at a scan rate of 100 mV/s is similar to that of the same type of material prepared by the co-precipitation method by GUO [21], exhibiting significant and symmetric oxidation and reduction peaks, which indicates the good reversibility of its electrochemical reaction process. The series of CV curves of the HEO2 sample also show clear and symmetric current peaks (indicated by the solid line), confirming the reversible redox reactions among the multiple metal ions in the electrode material [22,23]. Considering the interference of the water solutions’ decomposition current signal when the voltage exceeds 0.5 V, the test data were only analyzed in the range below 0.5 V.

From the perspective of kinetic characteristics, as the scan rate increases from 10 mV/s to 100 mV/s, the area of the oxidation/reduction peaks of the HEO2 sample increases linearly, and the areas of the two peaks are almost equal, which indicates that the transfer rates of electrons and ions in the electrode reaction process are relatively fast [24]. It is worth noting that due to the presence of polarization effects, the oxidation peak shifts towards higher potentials and the reduction peak shifts towards lower potentials. However, even at a high scan rate of 100 mV/s, the CV curve of HEO2 still maintains distinct peaks, demonstrating excellent rate performance [25,26].

Compared with the CV curves of HEO1, the oxidation peak potential of HEO2 is more negatively shifted, revealing its unique electronic structure characteristics: when elements such as Ga and Ge substitute for Fe and Mn, the d-band electronic structure of the electrode material is modulated, reducing the overpotential of the oxidation reaction and making HEO2 more prone to oxidation than HEO1. This potential shift reflects the enhanced catalytic activity of the material towards oxidation reactions, implying that in practical electrochemical reactions, HEO2 can more efficiently facilitate charge transfer, reduce energy loss, and thus improve energy utilization efficiency.

**Figure 6:**
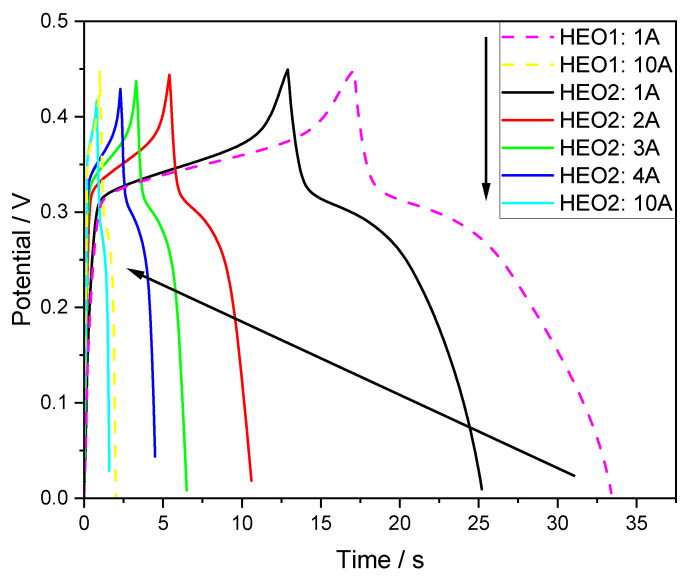
GCD curves of HEO1 and HEO2 electrodes at different current densities.

The galvanostatic charge–discharge (GCD) curves in Figure 6 show two distinct voltage plateaus: the charging plateau within the range of (0.325–0.375) V corresponds to the oxidation process in the electrochemical reaction, while the discharging plateau in the 0.275–0.325 V range corresponds to the reduction process. This is highly consistent with the redox potentials recorded in the cyclic voltammetry (CV) curves, indicating that the electrode material undergoes reversible redox reactions during the charge–discharge process with stable reaction kinetics. Dynamic changes in the GCD curves show that as the current density increases from 1 A/g to 10 A/g, the discharge time decreases nonlinearly, reflecting differences in the utilization rate of active material in the electrode at varying current densities.

**Figure 7:**
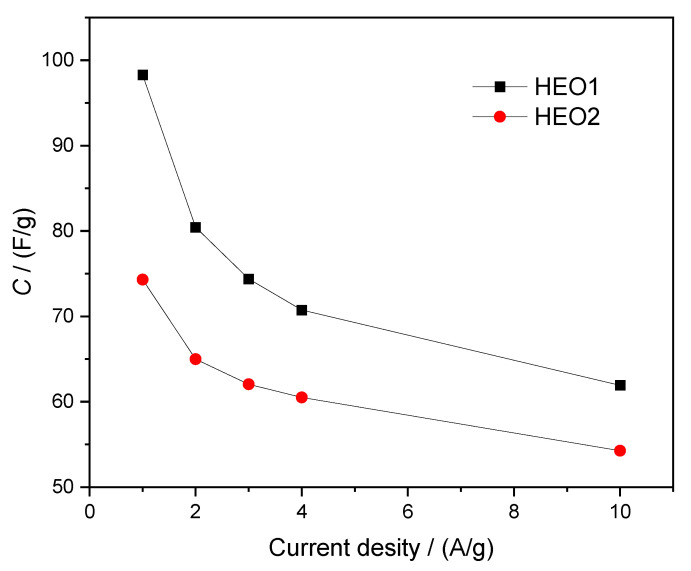
Comparison of specific capacities of HEO1 and HEO2 high-entropy oxide ceramics at various current densities.

The specific capacitance [27] of the electrode materials, calculated from the GCD data, is presented in Figure 7. The results show that the specific capacitance of the HEO1 electrode is 98.3 F/g at a current density of 1 A/g, and when the current density is increased to 10 A/g, the capacitance retention rate is 63% (61.9 F/g), which is lower than that prepared by the chemical method, but the capacitance retention rate is relatively good [21]. The specific capacitance of the HEO2 electrode is 74.3 F/g at 1 A/g, and when the current density is raised to 10 A/g, the capacitance retention rate reaches 73 % (54.3 F/g). Although the initial specific capacitance of HEO1 is 32.3% higher than that of HEO2, HEO2 exhibits better rate performance at high current densities, with a capacitance retention rate 10% higher than that of HEO1. This difference is attributed to the fact that HEO2 material may have better ion transport pathways, lower charge transfer resistance, or a more stable electrode structure, enabling it to maintain higher reaction efficiency during fast charge-discharge processes.

## 4. Conclusions

In this study, perovskite-structured high-entropy ceramic powders of La(${\mathrm{Co}}_{0.2}$${\mathrm{Cr}}_{0.2}$${\mathrm{Fe}}_{0.2}$${\mathrm{Mn}}_{0.2}$${\mathrm{Ni}}_{0.2}$)$O_{3}$ and La(Co_0.2_Cr_0.2_Ni_0.2_Ga_0.2_Ge_0.2_)O_3_ were prepared via solid-state synthesis processes at 1300 °C. Both high-entropy oxide ceramics exhibit a single-phase perovskite structure with uniformly distributed particles and result in a relative density exceeding 97%. Notably, the grain size of La(Co_0.2_Cr_0.2_Ni_0.2_Ga_0.2_Ge_0.2_)O_3_ is significantly larger than that of the counterpart. The working electrode fabricated by La(Co_0.2_Cr_0.2_Ni_0.2_Ga_0.2_Ge_0.2_)O_3_ as the electrode material shows higher oxidation reaction activity in CV measurements. In GCD tests, it delivers a specific capacitance of 74.3 F/g at a current density of 1 A/g and maintains 73% (54.3 F/g) of the initial capacitance when the current density increases to 10 A/g. Its capacitance retention at high current densities is 10% higher than that of La(Co_0.2_Cr_0.2_Fe_0.2_Mn_0.2_Ni_0.2_)O_3_, demonstrating superior rate performance.

## Data Availability

The original contributions presented in this study are included in the article/Supplementary Material. Further inquiries can be directed to the corresponding authors.

## Supplementary Materials

The following supporting information can be downloaded at: https://www.mdpi.com/article/10.3390/ma18173966/s1, Figure S1: Mapping results of Cr; Figure S2: Mapping results of Co; Figure S3: Mapping results of Ni; Figure S4: Mapping results of Ga; Figure S5: Mapping results of Ge.
