# Supplementary material for: Enhanced Rate Capability in B-Site High-Entropy Perovskite Oxide Ceramics: The Case of La(Co0.2Cr0.2Ni0.2Ga0.2Ge0.2)O3
Source: Materials (Basel). 2025 Aug 25;18(17):3966. doi: 10.3390/ma18173966 (PMC12429518; doi:10.3390/ma18173966)
Supplement: Supplementary file 1 [file materials-18-03966-s001.zip › materials-3768421-supplementary.pdf]

Supplementary Results

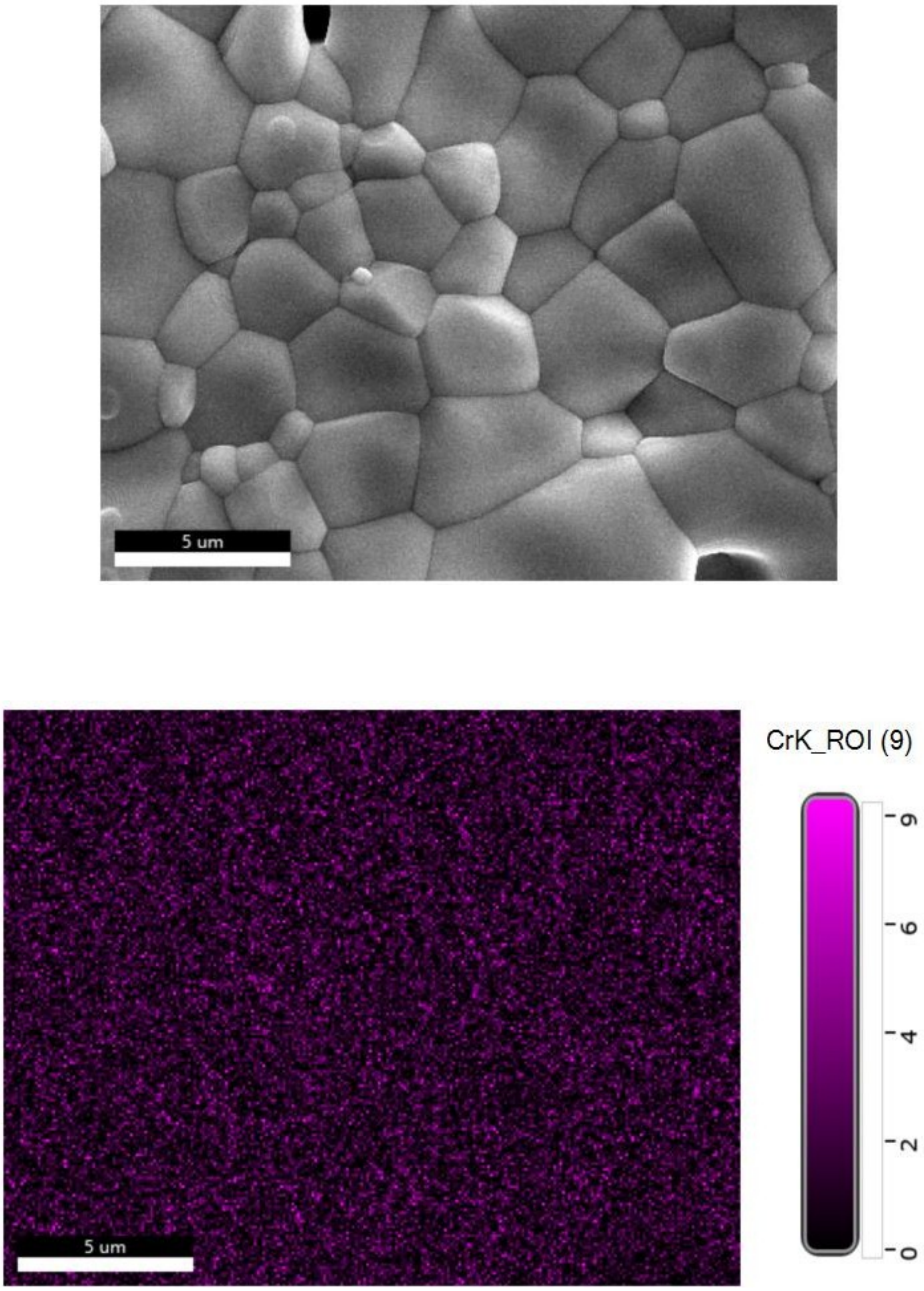

Figure S1   Mapping results of Cr

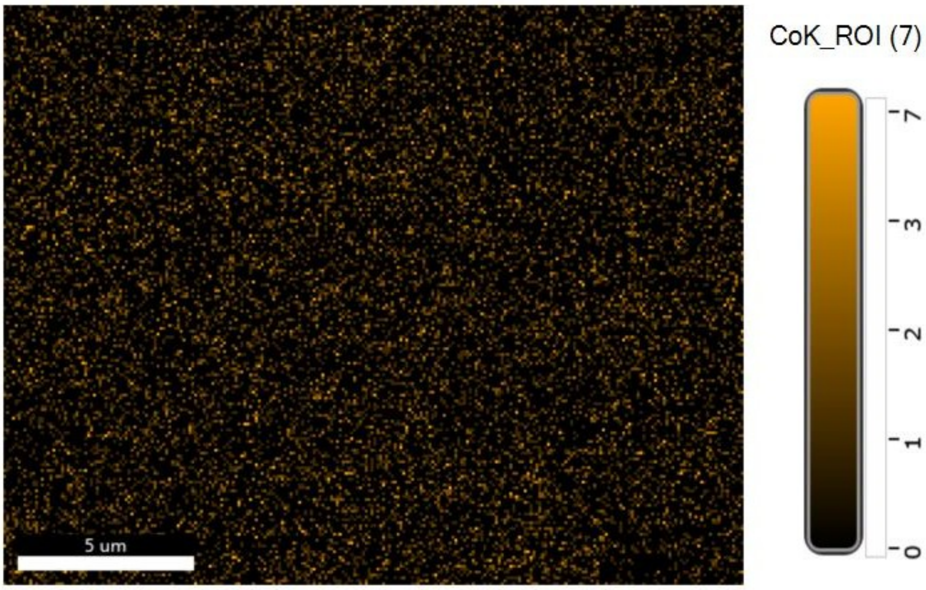

Figure S2 Mapping results of Co

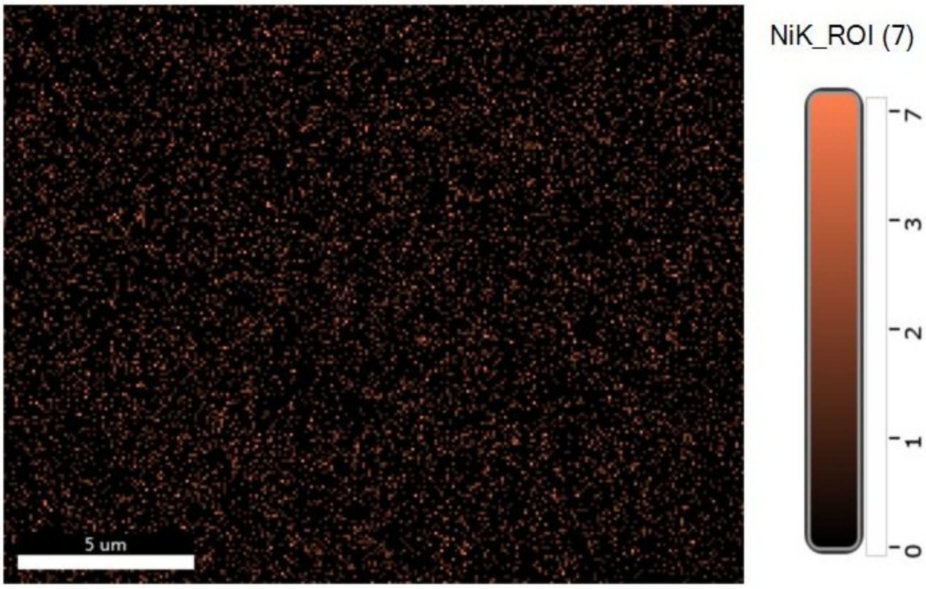

Figure S3 Mapping results of Ni

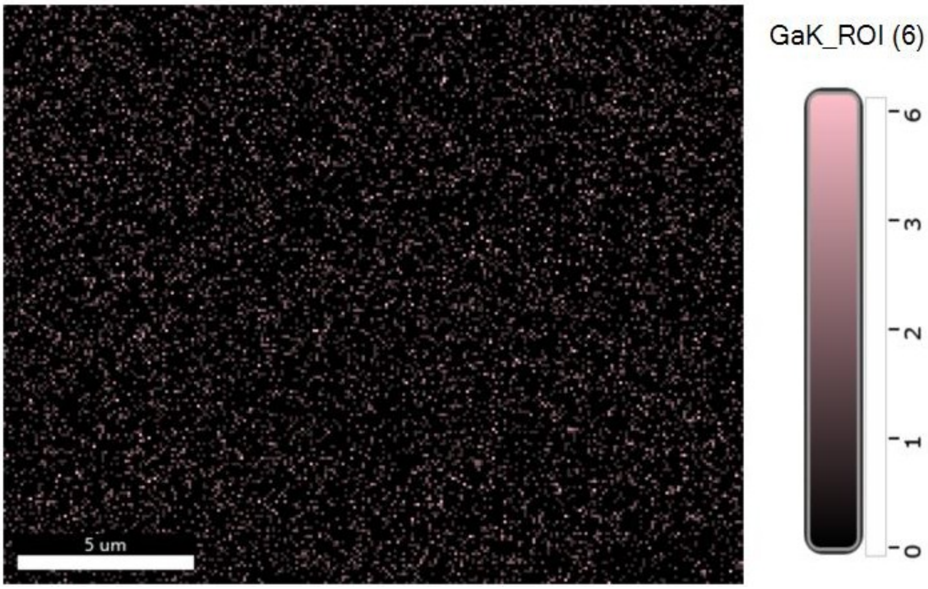

Figure S4 Mapping results of Ga

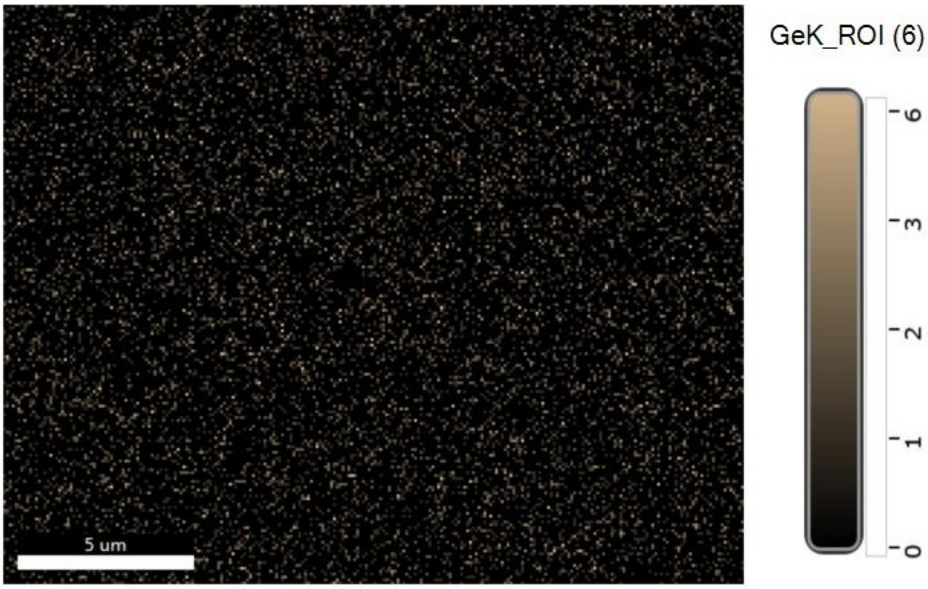

Figure S5 Mapping results of Ge
